# Acupuncture for drooling in children with cerebral palsy: A protocol for systematic review

**DOI:** 10.1097/MD.0000000000025393

**Published:** 2021-04-09

**Authors:** Wei Xiong, Ling Cheng, Genhua Tang, Xinju Hou, Manhua Zhu, Lunbin Lu, Zhiying Zhong

**Affiliations:** a Nanchang Hongdu Hospital of Traditional Chinese Medicine; b Jiangxi University of Traditional Chinese Medicine, Nanchang, PR China.

**Keywords:** acupuncture, cerebral palsy, children, drooling, protocol, systematic review

## Abstract

**Background:**

The aim of this study is to provide the methods used to evaluate the effectiveness and safety of acupuncture therapy for treating drooling in children with cerebral palsy.

**Methods and analysis:**

A comprehensive search of Pubmed, Embase, Cochrane Central Register of Controlled Trials, Web of Science, 4 Chinese databases (China National Knowledge Infrastructure, Chinese Biomedical Literatures database, Wan-Fang Database and Chinese Science and Technology Periodicals will be conducted to identify randomized controlled trials of acupuncture for treating children with cerebral palsy salivation with no restriction on time or language. The primary outcome of this systematic review will be the effective rate. The risk of bias will be implemented according to Cochrane Handbook for Systematic Reviews of Interventions. We will conduct the meta-analysis to synthesize the evidence for each outcome, if possible. The heterogeneity will be evaluated statistically using the *χ*^2^ test and the *I*^2^ statistic. The random-effect model will be used to provide more conservative results, if significant heterogeneity is identified (*I*^2^ > 50% or *P* < .10).

**Ethics/dissemination:**

Our findings will be disseminated in a peer-reviewed journal and at conference meetings. It is not necessary for formal ethical approval as no primary data are collected.

**Trial registration number:**

INPLASY2020110024

## 1 Introduction

Cerebral palsy (CP) is the most common neurological disorder, associated with drooling in childhood.^[1,2]^ Drooling indicates an upset in the neuromuscular incoordination control mechanism of swallowing leading to too much saliva in the anterior mouth and causing involuntary saliva to drain out of the mouth.^[3,4]^ It is approximated that 15% to 55% of children with CP have quoted the prevalence of drooling.^[3–5]^ In addition to health issues such as skin rashes, poor oral hygiene, and ulcers around the mouth, drooling causes significant on their daily socialization embarrassment, integration into society, and interpersonal relationships for individuals with disabilities.^[6,7]^

Surgery, pharmacologic treatments, botulinum toxin, physical, oro-motor and oro-sensory therapies, behavioral intervention, intraoral appliance, acupuncture are common treatments for constipation.^[7–15]^ They induce a considerable improvement in saliva flow and have been widely used all over the world. Nevertheless, they can also lead to side effects such as diarrhea, extreme dry mouth, loss of taste sensation, vomiting, and insomnia.^[16]^ More and more patients with drooling in children with CP are not satisfied with current treatment options. Acupuncture is gaining increasingly popularity as an alternative therapy for drooling in children with CP.^[17,18]^

Acupuncture, as an essential part of complementary medicine, has been a primary therapeutic method in China for more than 3000 years, and now its safety and effective treatment for various diseases in western countries.^[19]^

Traditional Chinese medicine (TCM) including acupuncture is based on the theory of Yin–Yang, qi, vital energy (qi) flows throughout the body by meridian passages. When there is harmonized dynamically imbalance, it will get sick. Stimulation of precisely defined point is believed to correct disruptions in harmony and to harmonizes functioning of the Zang-Fu organs.^[20]^ The practice of acupuncture is an effective treatment that is characterized by the insertion of inserting fine, solid needles (usually diameter, 0.25 mm; length, 40 mm) at locations on the body, which aims to adjust the imbalance in the flow of qi in meridians or “channels of energy flow.”^[21,22]^ Acupuncture is widely used in treatment various diseases, such as facial paralysis, cervical spondylosis, cerebral apoplexy, and drooling in children with CP.^[23,24]^ Its safety and efficacy of acupuncture treatment have been illustrated in several randomized controlled trials (RCTs).^[14,25]^ One study found that acupuncture can provide alleviate drooling in children with CP significantly and promote the child's brain development, improve the coordination of its central brain effectively.^[25]^ Another study found that tongue acupuncture is an effective treatment with the longstanding TCM concept to reduce their drooling dysfunction severity in children with CP.^[26]^ However, acupuncture for drooling in children with CP has not been reviewed systematically. It is worthy to define the effectiveness of acupuncture for drooling in children with CP to inform clinical practice.

### 1.1 Objectives

This systematic review aims to include RCTs to assess the evidence for the effectiveness and safety of acupuncture intervention for drooling in children with CP.

## 2 Methods

Study registration our systematic review protocol is registered in INPLASY 2020 (10.37766/inplasy2020.11.0024; https://inplasy.com/inplasy-2020-11-0024/). The protocol is reported following the recommendations of the preferred reporting items for systematic reviews and meta-analysis protocols statement guidelines.^[27]^

### 2.1 Criteria for considering studies for this review

#### 2.1.1 Types of studies

RCTs of acupuncture therapy for children with CP without publication type restrictions or any language will be included. Quasi- RCTs will be excluded as they are not truly randomized.^[28]^

#### 2.1.2 Type of participants

Participants of any race or gender who meet the diagnostic criteria of children with CP will be included. Patients should be between 2 and 17 years old.

#### 2.1.3 Types of interventions

Different types of acupuncture therapy (body acupuncture, dry needling, fire needling, electroacupuncture, warm needling, pricking-cupping, auricular acupuncture, catgut embedding, point injection) will be included in the review, regardless of the duration and frequency of treatment. Laser acupuncture, moxibustion and cupping will be excluded.

The control interventions could be drugs, physical activity therapy, herbs or herb extracts, western medicine, no treatment, placebo/sham acupuncture, or other interventions (eg, usual care, physical therapy). If the control group can carry out an acupuncture test, the comprehensive treatments containing acupuncture will be included, such as acupuncture plus another treatment compared with another treatment alone. Besides, trials to compare the effect of different kinds of acupuncture stimulations or different acupoints will be excluded.

### 2.2 Types of outcome measures

#### 2.2.1 Primary outcomes

We will assess the primary outcome by using the teacher drooling scale (TDS).^[16]^ We will indicate degree of drooling by TDS score, arranged from 1 to 5 points, where the higher the score of TDS, the drooling is more serious. Doctors, parents, or caregivers will evaluate TDS scores by the child's daily performance.^[5]^

#### 2.2.2 Secondary outcomes

The secondary outcomes will be measured through fluid volume, salivation frequency, and adverse events.

### 2.3 Search methods for identification of studies

#### 2.3.1 Electronic searches

We will search the following electronic bibliographic databases from their inception to January 2021: EMBASE, MEDLINE, Web of Science (science and social science citation index), Cochrane Library, Chinese National Knowledge Infrastructure, Technology Periodical Database, the Chongqing VIP Chinese Science, Wanfang Database, and Chinese Biomedical Literature Database, regardless of publication status, date and languages. The search terms will consist of 3 parts: drooling (e.g., “salivate,” “salivation,” or “drivel”), CP (e.g., “cerebral palsy” or “brain paralysis” or “spastic cerebral palsy”), acupuncture (e.g., “fire needling,” or “point injection,” or “electroacupuncture,” or “needle acupuncture”) and RCT (e.g., “randomised controlled trial,” “comparative study,” “prospective study,” “randomised controlled”). The search strategy for PubMed is shown in Table 1. The terms will be modified for other databases if necessary.

**Table 1:** Search strategy for the PubMed database.

Number	Search terms
#1	MESH: “children with cerebral palsy”
#2	Ti/Ab: “children with cerebral palsy” OR “children with CP” OR“children with brain paralysis”
#3	#1 OR #2
#4	MESH: “drooling”
#5	Ti/Ab: “drooling” OR “sialorrhea” OR “salivate” OR “salivation ” OR “drivel ”
#6	#4 OR #5
#7	MESH: “acupuncture”
#8	Ti/Ab: “acupuncture” OR “acupuncture therapy” OR “acupuncture points” OR “acupoints” OR “body acupuncture” OR “scalp acupuncture” OR “ auricular acupuncture” OR “ ear acupuncture ” OR “manual acupuncture” OR “electroacupuncture” OR “electro-acupuncture” OR “fire needling”
#9	#7 OR #8
#10	MESH: “randomized controlled trial”
#11	Ti/Ab: “randomized controlled trial” OR “RCT” OR “ controlled clinical trial” OR “ randomised ” OR “ randomly ”
#12	#9 OR #10
#13	#3 AND #6 AND #9 AND #12

### 2.4 Data collection and analysis

#### 2.4.1 Selection of studies

**Figure 1:**
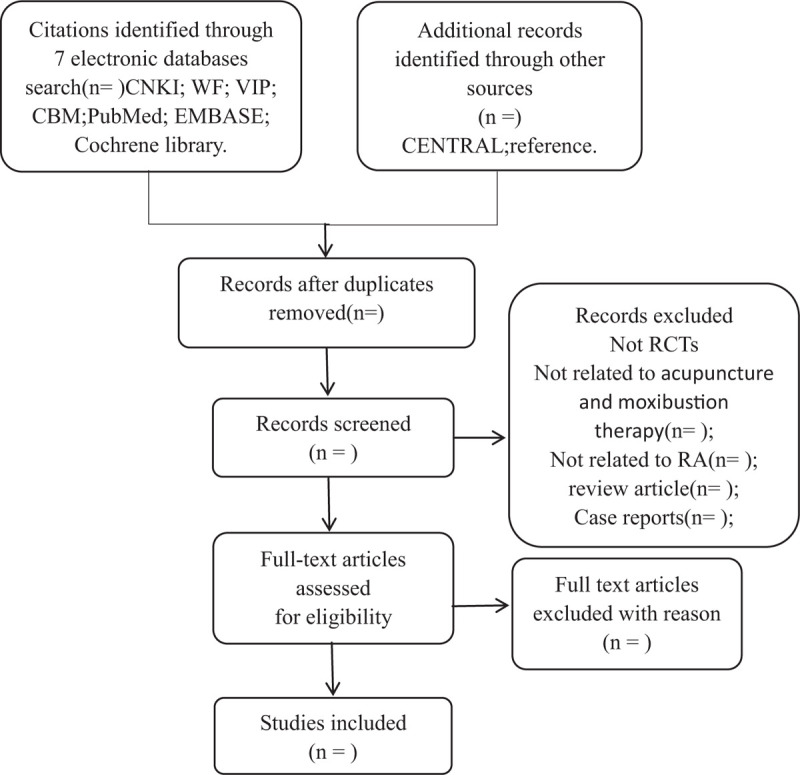
Flowchart of literature selection.

The titles and abstracts of all searched studies will be read after excluding the duplicated and irrelevant studies to determine the final included trials and separately critique all the identify eligible trials apparently. The above selection flow diagram will be shown in the preferred reporting items for systematic reviews and meta-analysis flowchart (Fig. 1).

### 2.5 Data extraction

Data for the RCTs will be screened by 2 independent review authors (ZSY and ZZY) using a piloted data extraction form according to the inclusion and exclusion criteria. The following information will be included.

(1) Study methods and characteristics (inclusion/exclusion criteria, method of randomization, blinding, withdrawals/dropouts, etc).
(2) Participants (number of participants, age range, gender, diagnostic criteria, disease course, disease course inclusion and exclusion criteria withdrawals/dropouts, etc).
(3) Intervention (type of acupuncture therapy, frequency, duration, etc).
(4) Control (no treatment, drugs, sham acupuncture, etc).
(5) Outcomes (reported outcomes, observation time points, adverse events, etc).

Any disagreements over data extraction will be consulted for consensus with a third experienced reviewer (LLB). We will transfer the new data into Review Manager Software 5.4.

### 2.6 Quality assessment

Included RCTs will be assessed the risk of bias by 2 trained research members (ZZY and TGH) using the Cochrane Risk of Bias Tool.^[29]^ This tool has 7 specific domains (blinding of participants, allocation concealment, random sequence generation, blinding of participants and outcome assessors selective reporting, incomplete outcome data, and other bias). Each domain is divided into 3 levels: low, high and unclear risk of bias. Blinding is often not possible in acupuncture therapy. Therefore, categorizing the performance bias in the RCTs as high risk of bias does not necessarily imply that the trial was poorly designed. Any inconsistencies will be resolved through consult with a third reviewer (LBL).

### 2.7 Measures of treatment effect

For continuous data (e.g.. TDS scores), mean difference (MD) with the corresponding 95% confidence intervals (CIs) will be used for analysis. For dichotomous data (e.g., adverse events), the risk ratio with the corresponding 95% CIs will be used for analysis. The standardized MD with 95% CIs will be used, if different scales were used to measure a certain outcome variable.

### 2.8 Unit of analysis issues

The unit of analysis will be based on aggregated outcome data as individual patient data is not be used for any research.

### 2.9 Missing data

The researcher will try to contact with the corresponding author to obtain the missing information. If the missing data are unobtainable, we will analyze only the available data. We will base our analysis analyze only the available data without making assumptions or imputing data. The potential impact of missing data from the review will be evaluated if necessary.

### 2.10 Assessment of heterogeneity

We will assess the heterogeneity by using the *χ*^2^ test or *I*^2^ value. According to the Cochrane Handbook, if the *P*-value of less than 0.10 in *χ*^2^ test or *I*^2^ is >50%, the heterogeneity across studies will be considered significant.^[30]^ We will use a random effects model to estimate overall treatment effect, if significant heterogeneity is identified (*I*^2^ > 50% or *P* < .10). Moreover, if the heterogeny remains significant, we will use a meta-regression and a subgroup analysis to explore the sources of heterogeneity among results of studies.

### 2.11 Assessment of reporting biases

If the studies included are more than 10 studies, we will use funnel plot to measure reporting bias.

### 2.12 Data synthesis

If possible, a random-effect meta-analysis will be analyzed and synthesized by Review Manager V.5.4. The purpose of this review, we will categorize outcome measures in 3 or more sections of assessment depending on the common follow-up time points in studies: immediate (within 3 weeks of the intervention delivery), short-term (3–10 weeks after intervention delivery), medium-term (11–15 weeks after intervention delivery), and long-term effects (15 or more weeks after intervention delivery).

If outcome measure scales are the same, we will use 95% CI to calculate the MD for continuous data. If outcome data are measured using different scales, we will use the standardized mean difference with the 95% CI.

When conducting the meta-analysis, *I*^2^ statistics will be used to assess the heterogeneity. According to the Cochrane Handbook, *I*^2^ < 50% will be considered as not important heterogeneity, we will consider heterogeneity to be substantial if *I*^2^ ranges from 50% to 90% and to be considerable if *I*^2^ ranges from 90% to 100%. If there is significant and considerable heterogeneity, we will explore its potential sources in sensitivity and subgroup analyses based on the type of the disorder.

If meta-analysis not possible, a narrative synthesis of the results will be presented, and quantitative findings for each study will be summarized descriptively.

### 2.13 Subgroup analysis

If data are available, we will perform subgroup analysis based on the type of intervention, different interventions of the control group and kind of different outcomes to interpret the heterogeneity.

### 2.14 Sensitivity analysis

Sensitivity analysis will be performed to assess whether the robustness of the study conclusions, assessing the impact of methodological quality, study design, the strength of evidence, sample size and the effect of missing data impact the results of this review. Furthermore, the *χ*^2^ test and *I*^2^ value will be performed to quantify statistical heterogeneity.

## 3 Discussion

Drooling is one of the most common complications for children with CP.^[31]^ According to TCM theory, drooling in children with CP is mainly caused by obstruction of meridians and collaterals, blockage of qi and blood, weakness of temper and loss of meridians’ nourishment.^[32]^ Patients with drooling have yin deficiency; alternatively, the drooling is also easy to consume the body's qi and yin, disrupting the balance of qi and blood. Lastly, this imbalance can result in disordered production and metabolism of body fluid. Although there are many treatment options, include posture correction, the use of anticholinergics, swallowing therapy, botulinum toxin injection and surgical treatment, adverse effects such as restlessness, blurred vision because of pupillary dilation, somnolence, xerostomia, confusion, decreased perspiration, and other adverse events.^[1,10,27,33]^

According to the theory of meridians, invisible pathways, are believed to constitute channels links together all parts of internal organs and also values the harmony between the heaven and body. The physiologic basis of TCM medicine is aimed to improve the physical condition by acupuncture treatment. Multiple studies have shown that acupuncture can obtain deqi (qi arrival), by the stimulation of specific points on the body, and even could take the place of drugs, with simple and clean efficiency, broad applicability.^[19,34–37]^

This systematic review will provide relatively reliable evidence of whether acupuncture therapy is effective for drooling in children with CP. This study will be the 1st SR of acupuncture treatment for drooling in children with CP. The process of conducting this review will be divided into 4 parts: identification, study inclusion, data extraction and data synthesis. The conclusions drawn may provide a systematic and comprehensive evaluation that will benefit patients, clinicians and makers health policy. Finally, we sincerely hope that the results of this study will offer the clinical recommendation for the clinicians and encourage more comprehensive application of acupuncture for drooling in children with CP.

## Author contributions

**Conceptualization:** Wei Xiong, Genhua Tang.

**Data curation:** Lunbin Lu, Xinju Hou, Manhua Zhu.

**Formal analysis:** Lunbin Lu, Manhua Zhu.

**Investigation:** Genhua Tang.

**Methodology:** Wei Xiong, Zhiying Zhong, Xinju Hou.

**Software:** Xingchen Zhou, Genhua Tang.

**Supervision:** Xinju Hou, Ling Cheng.

**Writing – original draft:** Genhua Tang, Xinju Hou.

**Writing – review & editing:** Genhua Tang, Lunbin Lu.
